# Particle filtration efficiency measured using sodium chloride and polystyrene latex sphere test methods

**DOI:** 10.1038/s41597-022-01860-y

**Published:** 2022-12-07

**Authors:** Timothy A. Sipkens, Joel C. Corbin, Andrew Oldershaw, Gregory J. Smallwood

**Affiliations:** Metrology Research Centre, National Research Council Canada, Ottawa, Canada

**Keywords:** Biomedical engineering, Characterization and analytical techniques

## Abstract

Standards governing face masks differ in the test methods used to determine sub-micron particle filtration efficiency (PFE), such that the meaning of PFE is not universal. Unifying the meaning of PFE requires data using these different test methods to drive improvements in standards. This simple data set provides the equivalence between two major test methods used to assess PFE: (1) a test method using a neutralized, polydisperse sodium chloride (NaCl) and (2) a test method using an unneutralized, “monodisperse” polystyrene latex sphere (PSL) aerosols. Measurements are made on over 5800 real-world medical masks, leading to the establishment of a relationship between these two kinds of PFE for these products.

## Abstract (summary)

**Table:** 

Measurement(s)	particle filtration efficiency
Technology Type(s)	TSI 8130 A • Palas PMFT 1000
Factor Type(s)	filtration media

## Background & Summary

The particle filtration efficiency (PFE) is a key performance characteristic of masks, face coverings, and related products that acts as personal protective equipment (PPE) for industrial health and safety, health care workers, and the general public. Numerous standards are available for these products, often varying by geographic region and product type. The recent pandemic has spawned research, raised public awareness of PPE, and highlighted some of the gaps in PPE standards, including inconsistencies in both how the PFE is determined and the recommended minimum PFE across the different standards. A summary of the key parameters in some of these standards is provided in Table 1.

**Table 1:** Specifications of products available for face masks and coverings using either (1) polydisperse NaCl with a CMD around 75 nm and (2) monodisperse PSL with CMDs around 100 nm.

Certification	Performance standard	Country of origin	Particle material	Particle size, CMD [nm]	Polydispersity, GSD	Particle size, MMAD [nm]	Measurand	Neutralization required?
N95	US 42 CFR, Part 84	USA	NaCl	75 ± 20	≤1.86	380 ± 95^†^	Mass	Yes
KN95	GB2626-2019	China	NaCl	75 ± 20	≤1.86	380 ± 95^†^	Mass	Yes
CAN95	CSA Z94.4.1	Canada	NaCl	75	≤1.86	380^†^	Mass	Yes
BFC	ASTM F3502-21	USA	NaCl	75	≤1.86	380^†^	Mass	Yes
-	ISO 16900-3:2012	International	NaCl	[60, 100]	[1.4, 1.8]	[150, 450]	Mass	Yes
FFP^‡^	EN 149:2001 + A1:2009	Europe, UK	NaCl	~66^‡^	~2.15^‡^	600	Mass	No
FDA Surgical Mask	ASTM F2299/F2100	USA	PSL	100	~ 1.0	100	Counts (~mass)	No

CMD is the count median *mobility* diameter. GSD is geometric standard deviation (with respect to mobility diameter). MMAD is the mass median *aerodynamic* diameter, computed from the expected density (spherules with a density of 2,160 kg/m^3^ for NaCl and 1,000 kg/m^3^ for PSL) of the particles, the CMD, and the GSD. The ISO method corresponds to an international standard that can be adopted to certify respirators, with the reported quantities for the NaCl variant of that test method. “BFC” stands for barrier face covering. The [*a*, *b*] notation denotes an interval of potential values from *a* to *b*.

^†^Assuming a GSD = 1.86 (i.e., max. for a CMD of 75 nm). ^‡^The test method is identical for FFP1, FFP2, and FFP3 products, differing in terms of required PFE. For this performance standard, the MMAD is constrained rather than the CMD. The GSD is approximate, based on the specified range of particle sizes (i.e., assigning [20, 2000] nm to ± 3σ). The CMD is computed based on this GSD and the MMAD. Some ambiguity is present in the interpretation.

**Table 2:** Details for the *NaCl* and *PSL* data sets.

Test (aerosol particle)	NaCl	PSL
Closest performance standard	US 42 CFR, Part 84	ASTM F2299/F2100
PFE measurement		
Instrument	TSI 8130 A	Palas PMFT 1000
Measurement principle	Photometer (45°)	Optical particle counter
Flow rate [LPM]	60	60
Face velocity [cm/s]	10	10
Neutralized	Yes	No
Target CMD [nm]	75	100
Geometric standard deviation (GSD)	≤1.86	~1.0 (monodisperse)
Pressure resistance measurement (no particles)		
Instrument	TSI 8130 A	Sataton T438
Flow rate [LPM]	60	8

Note that pressure resistance was determined without the presence of the aerosol in both cases and differences primarily drive from differences in the flow rate specifically during the pressure measurement. CMD is count median mobility diameter.

In particular, there is a pressing need to understand the equivalence between test methods using different challenge aerosols. Within this body of standards, two of the most common challenge aerosols for measuring sub-micron PFE are:

1. A neutralized, polydisperse sodium chloride (NaCl) aerosol, having a geometric median mobility diameter of 75 nm and a geometric standard deviation (GSD, with respect to mobility diameter) not exceeding 1.86. This is the aerosol used by: US 42 CFR 84^1^, which is used to certify N95 respirators in North America; GB2626-2019, used for KN95 respirators in China; CSA Z94.4.1, used for CAN95 respirators in Canada; ASTM F3502, used for barrier face coverings in North America; in a practical sense, ISO 16900-3:2012; and approximately EN 149:2001 + A1:2009, used to certify FFP1, FFP2, and FFP3 respirators in Europe. Variants of the NaCl method, without the same size requirements, are also featured in Australian/New Zealander (AS/NZS 1716–2012), Korean (KMOEL-2017-64), and Japanese (JMHLW/214, 2018) standards, but with consideration of a larger range of sizes. The aerosol is typically characterized by upstream and downstream photometers. This test aerosol has also formed the basis for a number of recent research works, e.g.^2–8^,.
2. A roughly monodisperse polystyrene latex (PSL) aerosol having a mobility diameter of 100 nm. This forms the basis for the existing ASTM F2299/F2100 test method^9^ used to measure the sub-micron PFE. This method, as it is presented, is general. For example, the method includes but does not mandate aerosol neutralization, does not specify the size of the PSL particles, and allows for a range of face velocities. The ASTM specification for medical mask material and the US Food and Drug Administration (FDA) document for medical/surgical masks^10^ narrows this, specifying that the particles have a diameter of 100 nm. Typically, and as is the case in this work, the particles are not neutralized when using this method, with consideration to the FDA document. The aerosol is typically characterized using optical particle counters (OPCs). Comparably few research studies have considered this challenge aerosol – e.g.^2,8,11–14^, – with results being more variable, given the lack of neutralization^8^.

**Fig. 1:**
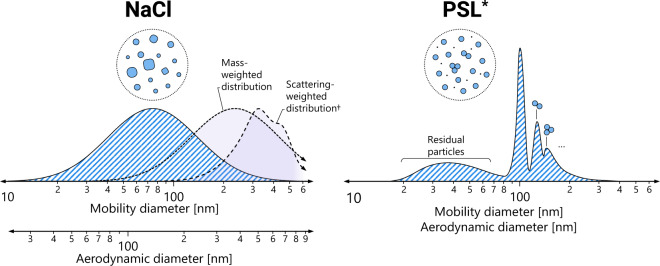
A schematic roughly demonstrating the size distribution for the two challenge aerosols considered in this work. The density of NaCl particles is substantially different than water, causing the mobility and aerodynamic diameters to be different for the same particle, indicated on stacked axes. By contrast, given that PSL particles are spheres having a density similar to that of water, the mobility and aerodynamic diameters of PSL are effective identical, indicated on a single axis. (*) The PSL distribution was chosen to match measurements by Corbin *et al*.^8^ (1 drop/mL), including the presence of doublets, triplets, etc. and residual particles. Hatched distributions are based on particle counts. (†) Solid purple-filled distribution in the left panel correspond to (long dash) the scattering-weighted distribution computed using Mie theory, representative of the photometer response in the NaCl test, and (short dash) the mass-weighted distribution, for which the scattering is considered an imperfect surrogate. The scattering-weighted distribution is adopted from ref. ^6^.

A schematic demonstrating these two challenge aerosols is given in Fig. 1. The PFE measured using these two aerosols are not equivalent, and converting between the variants is not intuitive^6^, requiring knowledge of the PFE resolved as a function of particle size. Standards organizations are working towards unifying the meaning of PFE. However, for this unification to occur, it is critical to have data that shows equivalencies between these different test methods for real-world products, where the current lack of data is significantly impacting standards development.

This work presents a significant amount of data where the PFE has been measured on real-world medical masks from a single manufacturer using the two methods above. This provides critical data in terms of the “equivalence” of these two test methods. This data supplements existing literature comparing other PFE test methods, for example incorporating super-micron bacterial filtration efficiency (BFE)^2,15^ or comparing number- and mass-based metrics for PFE^6,16–18^. Of particular note, Rengasamy *et al*.^2^ measured a series of samples using the NaCl (US 42 CFR, Part 84) and PSL (ASTM F2299) methods, analogous to this work. That work was limited to 12 lots but spanned a larger range of PFEs. This work supplements that work with substantially more data (over 600 lots corresponding to over 6,000 samples), and a focus on the medical mask PFE range.

## Methods

**Fig. 2:**
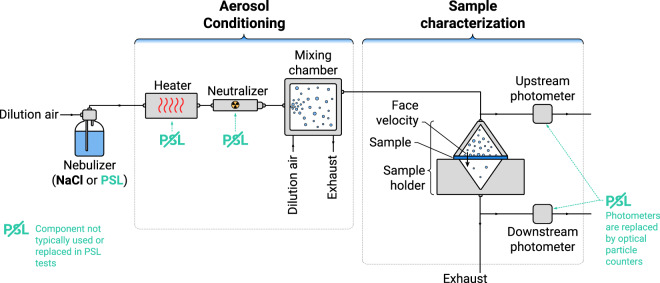
Schematic of a typical system used to measure particle filtration efficiency of a sample with an upstream and downstream photometer, similar to the TSI 8130 A and PALAS PMFT 1000 systems used in this work. Aerosol conditioning elements are representative of the NaCl test performed by the TSI 8130 A. For example, this setup has a neutralizer and detectors composed of photometers, whereas the PSL aerosol is typically not neutralized and uses an optical particle counter for characterization. Differences in the PSL test method are marked with teal annotations. For more details on the differences between the test methods, see Table 1.

Both the NaCl and PSL test methods make use of a similar apparatus, demonstrated schematically in Fig. 2. In both cases, an aqueous solution is nebulized to produce an aerosol laden flow. This flow is conditioned to control the aerosol characteristics, e.g., using a heater to isolate particles from the nebulizer water droplets and particle neutralization. The precise type of particle conditioning varies depending on the test method and instrument, e.g., the PSL test aerosol is typically not neutralized. The aerosol then passes through the test section, composed of an upstream detector, to measure the particles in the air ahead of the filter; a sample holder, with an opening of a specific size; and a downstream detector, to measure the particles after the filter. The NaCl test uses photometers as detectors, which measure the scattering from an ensemble of particles (at an angle of 45° from the forward direction of light propagation in the TSI 8130 A). This roughly assesses the mass concentration of aerosol in the particle stream (more specifically, a response integrated over the scattering-weighted distribution, which is a rough approximation^6^ and could be calibrated to mass^18^). This effectively shifts the challenge particle size to larger sizes, as is discussed elsewhere^6,17^ and demonstrated schematically in Fig. 1. By contrast, the PSL test uses an optical particle counter (OPC), which, while also based on light scattering, counts the particles by detecting pulses of light scattered from individual particles as they pass through a beam of light.

Results for the NaCl test here were obtained using a TSI 8130 A, in line with the relevant parameters of US 42 CFR 84 test method^1^, without consideration of the loading requirements, and the ASTM F3502 test method. Particles had a count median mobility diameter of 75 nm and geometric standard deviation (with respect to mobility diameter) not exceeding 1.86. The instrument aerosol size distribution was verified using Greenline media to assure that it was within the specifications. Reported results correspond to the *initial PFE*, in this case the PFE after only a minute of loading. As noted above, the TSI 8130 A uses photometers to measure the particle concentrations. The reported PFE values were taken from the instrument’s software for both cases, which is taken as a black box, as is typical of measurements gauging standards-compliance. This roughly corresponds to the ratio of the mass concentration of particles downstream to that upstream, such that the corresponding output of the TSI 8130 A can be considered a mass-based particle filtration efficiency^6^.

PSL results were obtained using a Palas PMFT 1000 and following the ASTM F2299 test method^9^. Particles were not neutralized, having the charge distribution imparted by the built-in nebulizer. The PSL used in this study had a nominal diameter of 100 nm, consistent with the FDA recommendations^10^. Number concentrations of particles are low enough that reported results can also be considered initial PFEs, in this case without any impact from loading. Measurements were made using optical particle counters, yielding count-based measurements and number-based particle filtration efficiency^6^. Note, however, that the PSL aerosol is theoretically monodisperse, such that the mass-based and number-based filtration efficiencies should be similar. As with the TSI 8130 A measurements, results were otherwise reported by treating the Palas instrument as a black box.

In both cases, samples were mounted to a plate in the instrument and were tested flat at a flow rate of 60 LPM, which corresponds to a face velocity of 10 cm/s for both instruments. For both test methods, five samples were measured from each lot. All of the samples were ASTM Level 3 candidates composed of non-woven polypropylene. The samples did not undergo any conditioning. Given the destructive nature of the test method (loading the samples with particles), the NaCl and PSL tests were not performed on the same samples. While all samples were from the same lot, this will result in sample-to-sample variability being convolved with differences between the tests, resulting in scatter.

For the NaCl data, the pressure resistance is measured using at a flow rate of 60 LPM. By contrast, for the PSL data, the pressure resistance is measured at a flow rate of 8 LPM. The naming of the two cases (NaCl and PSL) is only to align the measurements with the PFE measurement associated with a given data set, as particles were not present during pressure resistance testing. Of note, the flow rate was 60 LPM for both of the PFE tests.

## Data Records

The data is archived on figshare^19^.

Data is compiled into lots, each of which contains pairs of NaCl and PSL results. Data corresponds to quality control data and includes a total of 5820 samples across 582 lots, with five samples per method (NaCl and PSL) per lot. The individual sample data is stored in a JSON format, with one entry for each lot. For each lot, the data contains eight entries summarized in Table 3. PFEs are consistently reported as a percent, while pressure resistances are consistently reported in Pa. Lot-averaged data is also provided in a CSV format for faster processing, with two columns containing the PFE in percent as measured by the (Col. 1) NaCl/TSI 8130 A and (Col. 2) PSL/Palas tests.

**Table 3:** Data fields for the JSON formatted data^19^.

Field name	Description	Units	Field type
ID	Lot identifier	—	Integer
PFE_NaCl_ave	PFE for the NaCl test, averaged over 5 samples	%	Numerical
PFE_NaCl	PFE for the NaCl test for each sample	%	[5 × 1] numerical array
PFE_PSL_ave	PFE for the PSL test, averaged over 5 samples	%	Numerical
PFE_PSL	PFE for the PSL test for each sample	%	[5 × 1] numerical array
DP_NaCl_ave	Pressure resistance for the NaCl test (60 LPM), averaged over 5 samples	Pa	Numerical
DP_NaCl	Pressure resistance for the NaCl test (60 LPM) for each sample	Pa	[5 × 1] numerical array
DP_PSL_ave^†^	Pressure resistance for the PSL test (8 LPM), averaged over 5 samples	Pa	Numerical
DP_PSL^†^	Pressure resistance for the PSL test (8 LPM) for each sample	Pa	[5 × 1] numerical array

See Table 2 for more information on the NaCl and PSL methods.

^†^Note that the pressure resistance for the “PSL” data was determined at a different flow rate (8 LPM) in the Palas PMFT 1000. As such, the pressure resistance values are not directly comparable to those associated with the “NaCl” data.

## Technical Validation

The laboratory that made these measurements participated in an interlaboratory comparison (ILC)^20^ that covered a range of instruments, including other TSI 8130 A systems, PALAS systems, and custom-built systems based on particle counting (e.g., the PFEMS developed by the National Research Council Canada^8,21^). Each laboratory was instructed to execute a specific protocol, which included measuring the pressure resistance of a pre-characterized orifice plate, measuring the PFE and pressure resistance of filtration media with a range of specifically targeted level of PFE, and the PFE of a single lot of respirators. The laboratory was found to be within an acceptable range of the other laboratories that participated in that study across all of the test cases.

**Fig. 3:**
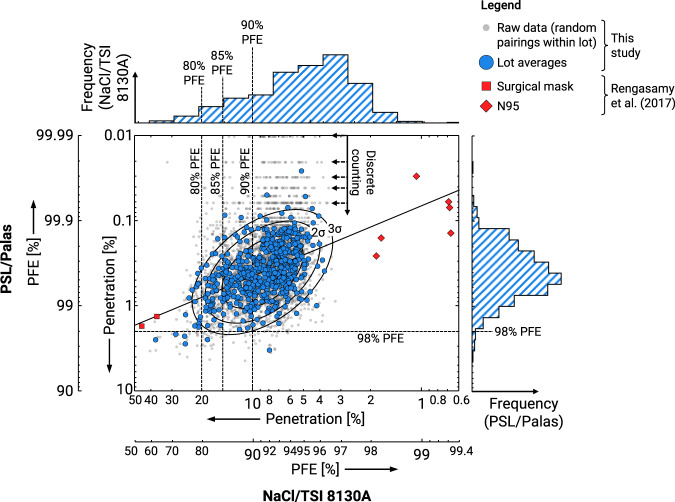
Comparison of the non-neutralized PSL and neutralized NaCl filtration efficiencies. Larger blue points correspond to lot-averaged data. Light grey points correspond to the underlying, raw, unaveraged data, shown to give a sense of the broader extent of data and demonstrate the presence of discrete counting artifacts in the PSL penetration measurements. Within lot pairings (light grey points) between the two methods were random, not samples that were tested via both the NaCl and PSL methods. These points are only intended to show the broader extent of the data, rather than correlation between the two data sets, and to demonstrate the discrete particle counting artifacts in the upper region of the plot. Solid, black ellipses correspond to covariance ellipses computed on the log of the data, and correspond to 1, 2, and 3 standard deviations when moving from the center to the outside. Histograms are indicated on either axis, showing the distribution of the lot-averaged data. Data points from Rengasamy *et al*.^2^ correspond to surgical masks and N95/surgical N95 respirators.

Figure 3 shows pairs of NaCl and PSL data from the current data set, alongside the 12 lots from Rengasamy *et al*.^2^ that span a larger range of PFEs. The Rengasamy *et al*. data lies about an extrapolation of the central trend of the data presented in this study. This provides further validation of the observations.

Beyond this validation, these are common instruments in this field that were used according to their instructions and were regularly calibrated according to the manufacturer specifications. As noted in the Methods section, these instruments are otherwise treated as black boxes, being consistent with the use of these instruments when gauging conformity to standards. Thus, after ensuring calibration of the instruments and proficiency of the laboratory, data is supplied *as is* in order to gauge the reliability of the standard test methods in their current state. Note that this means that the natural charge distribution produced by the nebulizer in the PALAS system is not explicitly characterized prior to measurement. Neutralization of the aerosol in the TSI 8130 A produces an aerosol with a known range of charges, considered more reliable across instruments and laboratories.

**Fig. 4:**
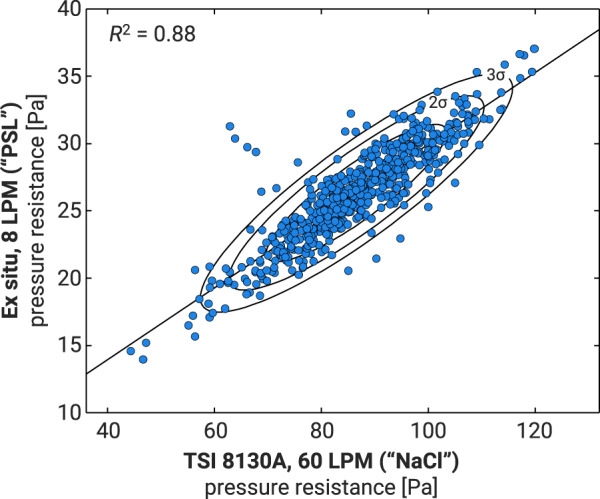
Lot-averaged pressure data associated with each of the data sets. The “NaCl” data is measured using a TSI 8130 A at 60 LPM. The “PSL” data is measured with a Sataton T438 at 8 LPM. The differences in pressure drop are expected to drive differences between the data sets. Solid, black ellipses correspond to covariance ellipses computed on the data, and correspond to 1, 2, and 3 standard deviations when moving from the center to the outside. The reported *R*^2^ value is the Pearson correlation coefficient between the two data sets.

As noted above, the pressure resistance was measured using different flow rates for the “NaCl” and “PSL” data. Figure 4 shows the lot-average pressure resistance pairs across the two data sets. The results are clearly shifted with respect to one another but remain correlated. The correlation suggests robustness in the measurements in that, if a mask has a high pressure resistance, this held true at both flow rates.

## Usage Notes

The primary function of this data is to support standards development, allowing for a comparison of two major test methods for a range of real-world, ASTM Level 3-candidate surgical masks. Secondarily, this data set is to give researchers insights into the real-world performance of filtration media designed for the market, including how different ways of measuring particle filtration efficiency can result in very different outcomes.

Log axes with respect to the penetration were chosen for Fig. 3. This is consistent with the way uncertainties are to be reported in the ASTM F2299 test method^22^, where GSDs are used for errors instead of standard deviations. Such a treatment is roughly based on two principles: (1) that as more layers are added to a product, the resultant penetration is the product of the penetration of the individual layers (which acts as a surrogate when the filtration media is thicker, as may occur during manufacturing), and (2) that data containing Poisson noise will result in a narrowing of the uncertainty in the PFE as the PFE increases^23^. Histograms indicate that the points are roughly normally distributed in log space, with some skew evident in the NaCl test data. Further, samples are roughly evenly spread above and below the central trend, providing support for this treatment.

Grey points in Fig. 3 correspond to unaveraged data, with data randomly paired within the lots. Given these random pairings, this data is only intended to demonstrate characteristics of the raw data, including their extent and the presence of discrete counting artifacts, rather than any correlation between the two test methods.

While there is a distinct shift in the PFE, there is substantial scatter about the central trend. Since the NaCl and PSL tests were not performed on the same sample, sample-to-sample variability is partially responsible for this scatter. This also suggests within lot variations and measurement fluctuations, rather than inter-lot variations, are likely a dominant source of uncertainty. One can also observe a higher degree of scatter in the individual (grey points in Fig. 3) PSL results relative to the NaCl results. This observation likely stems from day-to-day variations in the natural charge distribution imparted by the nebulizer in the PSL case, which is less controlled variable relative to the neutralized NaCl aerosol. The remaining trend in the penetration, generally consistent with Rengasamy *et al*.^2^, is given as a power law:

1 $${P}_{{\rm{PSL}}}={\rm{0}}{\rm{.0667}}\cdot {\left({P}_{{\rm{NaCl}}}\right)}^{0.828}$$

or, equivalently,

2 $${P}_{{\rm{NaCl}}}={\rm{26}}{\rm{.3}}\cdot {\left({P}_{{\rm{PSL}}}\right)}^{1.21},$$

where *P*_NaCl_ and *P*_PSL_ are the penetration with respect to the NaCl and PSL methods respectively (as a percentage).

Some caution is expressed regarding changes to the type of filtration material and its effect on differences in the measured PFE between these two test methods. Changes in the size-resolved PFE can cause structured differences. This is particular in light of potential innovation to filtration media away from the standard non-woven polypropylenes or when changing the charge state of the material.

As noted in Methods and in connection with Fig. 4, pressure resistance data are measured at different flow rates. As such, values are not directly comparable and should not be used as such.

This data has been provided anonymously by a mask manufacturer, the identity of which is not disclosed for reasons of commercial sensitivity. As such, some of the underlying parameters have not been made publicly-available. The data has been released under a Creative Commons license. There are no other restrictions on the data as it is presented in the associated repository.

## Data Availability

Beyond the raw data files, we have also included code in Python, JavaScript (via the Node.js framework), and Matlab to read the JSON file into a native format (e.g., a Python dictionary).
